# Effect of congenital color vision deficiency on the ability of optometrists to correctly identify lesions in ocular fundus photographs

**DOI:** 10.1371/journal.pone.0337626

**Published:** 2025-11-24

**Authors:** Richard C. Trevino, Anna A. Tichenor, Susan Kovacich, Hin Cheung

**Affiliations:** School of Optometry, Indiana University, Bloomington, Indiana, United States of America; L V Prasad Eye Institute, INDIA

## Abstract

**Purpose:**

To investigate the influence of color vision deficiency (CVD) on the ability of practicing optometrists to correctly identify lesions in digital color fundus photographs with and without the aid of a commercially available color vision remediation device.

**Methods:**

This study was conducted at the 2024 annual meeting of the American Academy of Optometry in Indianapolis, IN, USA. The color vision of each subject was assessed using the Konan ColorDx CCT-HD®. Individuals with a score <90 for any cone type were classified as having CVD. Each subject then attempted to correctly identify lesions in a series of 12 digital color fundus photographs. Subjects identified as having CVD repeated the photograph task while wearing EnChroma® indoor lenses. The quality of life (QoL) of each subject was evaluated using a modified version of the Color Blindness QoL survey.

**Results:**

Fifty-five optometrists completed the study. Forty had normal color vision (CVN) and 15 had CVD. Four of 15 (27%) CVD optometrists were previously unaware of their CVD. Both age and CVD influenced the ability of optometrists to correctly identify lesions. Among CVN subjects, younger clinicians (<39 years) outperformed older ones (p = 0.001). However, among subjects with CVD, age did not significantly affect performance (p = 0.84). Compared across CVD status, younger clinicians with CVD performed worse than their normally sighted peers (p = 0.002) while CVD had little effect among older clinicians (p = 0.23). Performance did not improve with use of the EnChroma lenses. Approximately half (47%) of optometrists with CVD reported difficulties in their daily activities attributed to poor color perception.

**Conclusions:**

CVD and older age decreased the ability of optometrists to correctly identify lesions in fundus photographs. Performance did not improve with use of the EnChroma lenses. Our findings suggest that CVD may pose a disability to eye care providers. We endorse the recommendation that individuals entering medical practice undergo color vision testing and counseling if a deficiency is found.

## Introduction

Approximately 8% of Caucasian males have some form of red-green color vision deficiency (CVD), and prevalence varies among other racial and ethnic groups [1]. Because red-green CVD is inherited as a sex-linked trait, the prevalence among females is extremely low. Approximately 0.5% of Caucasian females are reported to have CVD. There are several varieties of CVD [2]. Broadly, they may be classified as deutan, protan and tritan defects. Deutan defects are the most common (approximately 6% of males) and are associated with an absence of M-cone (green) photoreceptors or a shift in their spectral sensitivity towards the red. Protan defects are identified in about 2% of males and are associated with absent L-cone (red) photoreceptors or a shift in their spectral sensitivity toward the green. Tritan defects (S-cone, blue) are the least common (< 0.01% of individuals) and are inherited as an autosomal dominant trait. CVD is associated with wide-ranging detrimental effects on activities of daily living, especially in work-related activities [3,4], and hence may decrease the quality of life (QoL) of affected individuals. Studies using the Color Blindness Quality of Life (CBQoL) questionnaire have shown that individuals with CVD score significantly lower on measures of quality of life related to health, lifestyle, work, and emotions than those with normal color vision [5–7].

CVD can significantly impact clinical practice, affecting a medical professional’s ability to accurately perceive and interpret visual information [8]. Because individuals with defective color vision do not perceive colors normally, they may miss lesions or incorrectly identify them. Research studies have documented the adverse effect of CVD in general medical practice [8–11] but, to our knowledge, only one such study has specifically investigated the effect of CVD on the performance of eye care providers. AlRyalat et al., using photographs of diabetic retinopathy that were manipulated to simulate abnormal color vision, found that CVD resulted in decreased accuracy in assessing diabetic retinopathy and macular edema [12].

Various lenses and filters have been developed to help improve the color perception of individuals with CVD [13–15]. These devices work by attenuating a narrow band of the wavelengths of light entering the eye to increase color contrast or provide new cues for color discrimination. A recent systematic review and meta-analysis found that there is insufficient evidence to conclude that any commercially available device improves color vision perception [15]. To our knowledge, this is the first study to assess the impact of such a device on practicing optometrists’ performance in identifying retinal lesions.

The purpose of this study is to determine the extent to which CVD impacts the ability of practicing optometrists to correctly identify lesions in color photographs of the ocular fundus. We then investigated the effect of a remediation device on the performance of CVD optometrists in performing this task. Finally, we investigated the effect of CVD on the quality of life of optometrists using a validated survey instrument.

## Methods

This study was approved by the Institutional Review Board of Indiana University (IRB #23406) and was conducted in accordance with the principles of the Declaration of Helsinki. Subjects were recruited from the Fellows Doing Research booth in the exhibit hall at the 2024 meeting of the American Academy of Optometry in Indianapolis, IN, USA. Inclusion criteria were practicing optometrist at least 18 years of age with visual acuity of at least 20/30 in each eye. Subjects were excluded from participation if there was a history of any active or inactive ocular disease with the potential to impact visual function, and if screening with HRR pseudoisochromatic plates detected a CVD in one eye only or an anomalous/unclassified CVD.

All individuals expressing an interest in participating in the study completed a written informed consent document and a questionnaire that included demographic information and a modified version of the Color Blindness Quality of Life Survey (CBQoL). The CBQoL is a validated 23-question survey that investigates the impact of CVD in 3 domains: Health and Lifestyle, Emotions and Work [5]. The CBQoL was modified for our study by the addition of 3 questions that specifically targeted tasks of optometric practice. Visual acuity was assessed with a reduced Snellen chart at 40 cm using the subject’s habitual near or “computer-use” correction. Subjects with less than 20/30 acuity in either eye were excluded from the study.

Subjects who met our inclusion criteria underwent screening for CVD using the HRR (Hardy Rand and Rittler) 4^th^ edition pseudoisochromatic plates. The plates were displayed under a commercially available 5000 K light bulb in addition to the overhead lighting of the exhibit hall. Colorimetric testing using the MK350N Premium handheld spectrometer (UPRtek, Taiwan) revealed that the correlated color temperature at the HRR testing station was 5360 K and the color rendering index was reported as 83.7. Those subjects who passed the HRR screening or who exhibited a bilateral CVD that was classified by HRR as being either deutan, protan, or tritan in nature then underwent further testing with the ColorDx CCT-HD® system (Konan Medical, Irvine, CA) displayed on laptop computers provided by Konan. ColorDx is an implementation of the Rabin Cone Contrast Test [16,17]. In addition to reporting a score that is derived from the contrast threshold for each cone type, the ColorDx also reports the mean response time for each cone type, which Konan describes as a “secondary measure of performance.” Prior to use each of the 4 laptop computers used in this study were calibrated by Konan. The laptops were placed under a canopy to minimize the influence of overhead exhibit hall lighting. Testing was performed binocularly using the full-threshold strategy for all cone types (L, M and S) at a test distance of approximately 0.5m using software version 1.0.79. A ColorDx score of less than 90 for any cone type was considered evidence of CVD, as recommended by the ColorDx User Manual. When there was disagreement between HRR and ColorDx results, the ColorDx results were used.

Following color vision testing the subjects viewed a series of 12 digital color fundus photographs drawn from the authors’ personal collection. The images were of a variety of disorders, including images of hemorrhage, pigmentation, and optic nerve pallor (S1 File). While wearing their habitual near or “computer-use” correction subjects were asked to carefully inspect each photograph and to report any lesions that were detected. No clinical context (patient age, medical history, etc.) was provided. Subjects recorded their responses in a free-form text field. The task was not timed, and subjects were free to zoom images and to return to previously viewed images to edit their responses. The photographs were displayed on the same laptops that were used for the ColorDx test and were presented in the same sequence for all subjects.

Two graders (RCT and SK) independently scored subject responses as correct or incorrect for each photograph. A third grader (AAT) independently scored responses and served as tiebreaker when the initial graders disagreed. Graders were blinded to the color vision status of the subjects while evaluating their responses.

Subjects with CVD as determined by ColorDx repeated the fundus photograph task while wearing EnChroma® indoor remediation lenses. They were then asked to subjectively report whether the remediation device made it easier to identify lesions in the photographs.

Our primary outcome measure was the number of correctly identified photographs (photo score) by color vision normal (CVN) compared to CVD subjects. Secondary outcome measures were the effect of age and the remediation device on the correct identification of lesions and differences in CBQoL between CVN and CVD subjects. The Shapiro-Wilk test was performed to test data for normality. The Mann-Whitney U test was performed to evaluate performance across the entire set of photographs and the Pearson chi-square test evaluated performance on each photograph. Stepwise regression analysis was performed to identify those factors that were most influential on photo score. The Wilcoxon signed ranks test was performed to evaluate the effect of the remediation device on correct lesion identification across the entire set of photographs. Student’s t-test was performed to evaluate differences in ColorDx scores. The Pearson chi-square test was performed to compare CBQoL survey results of subjects with normal and abnormal color vision. Statistical analyses were performed using SPSS version 29 (IBM Corp., Armonk, NY).

## Results

A total of 93 subjects were recruited into the study. Eight subjects were excluded because they failed to complete the study protocol. One subject was excluded because a unilateral color vision defect was detected on HRR screening and 1 subject was excluded because their color vision defect was classified by HRR as ‘Other/Unclassified’. Therefore, data from a total of 83 subjects were available for analysis.

Of these 83 subjects, 21 listed their occupation as student, 5 as scientist, and 2 as other. These 28 subjects were also excluded from the final analysis. We were left with 55 subjects whose occupation was reported as optometrist or resident. Self-reported race of the subjects was n = 37 white, n = 14 Asian, n = 1 African-American, and n = 3 other/multiracial.

Of the 55 subjects included in the final analysis, 40 (73%) had normal color vision (CVN) as determined by ColorDx, and 15 (27%) were diagnosed with CVD. Among those with CVD, 15 failed on M-cone (green) testing, 10 failed on L-cone (red) testing, and 2 failed on S-cone (blue) testing. (Note: subjects may fail on testing of more than 1 cone type). Among those with CVD, 11 were aware that they had a CVD and 4 (27%) reported not being previously aware. Three of the 4 unaware individuals passed HRR screening but failed the ColorDx exam. The fourth unaware individual was a female optometrist that failed HRR screening with a mild deutan defect.

Eleven of 15 individuals who failed ColorDx also failed HRR screening. Classification of CVD per HRR was 8 deutan (1 strong. 4 medium, 3 mild) and 3 protan (2 strong, 1 mild) deficiency. There was no significant difference in photo task performance between optometrists with protan and deutan defects for any of the 12 fundus photos (Data not presented).

**Fig 1:**
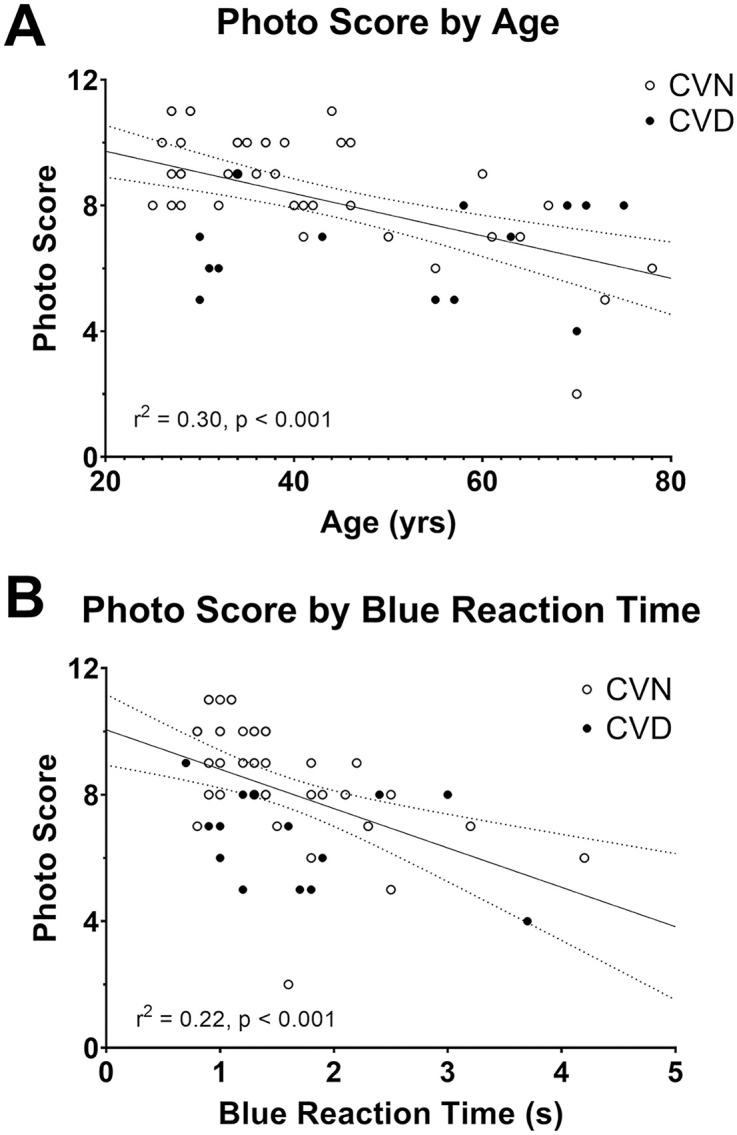
Scatter plots of photo score by age and blue reaction time. CVN: Color vision normal subjects, CVD: Color vision deficient subjects. Photo score: Number of correctly identified photographs.

Stepwise regression analysis was performed on our dataset of 55 optometrists to identify the most influential predictors of performance on the lesion identification task. The photo score was the dependent variable and the six ColorDx metrics (red, green and blue scores and reaction times) plus age were independent variables. The final model containing green score and blue reaction time was statistically significant (F(2,51) = 21.02, p < 0.001) and accounted for a substantial proportion of photo score variance (r^2^ = 0.45). In the first step, the regression equation was Score = 11.08 + (−0.67 * Age) indicating a significant negative influence of age (t = −4.64, p < 0.001). Age remained significant in the second step after green score was added to the model but was no longer significant after blue reaction time was added in the third step. The regression equation of the final model was Score = 7.36 + (0.03 * green score) + (−1.18 * blue time) indicating a significant positive contribution of green cone contrast sensitivity (t = 4.66, p < 0.001) and a significant negative influence of reaction time to blue stimuli (t = −4.23, p < 0.001). Age (r = −0.54, p < 0.001) and blue reaction time (r = −0.47, p < 0.001) were negatively correlated with photo score, and were strongly correlated with each other (r = 0.70, p < 0.001) (Fig 1). Reaction times to red (r = 0.51, p < 0.001) and green (r = 0.38, p < 0.001) stimuli were also significantly correlated with age, but not with photo score (red: r = −0.18, p = 0.19; green: r = −0.12, p = 0.37).

### Influence of age

**Fig 2:**
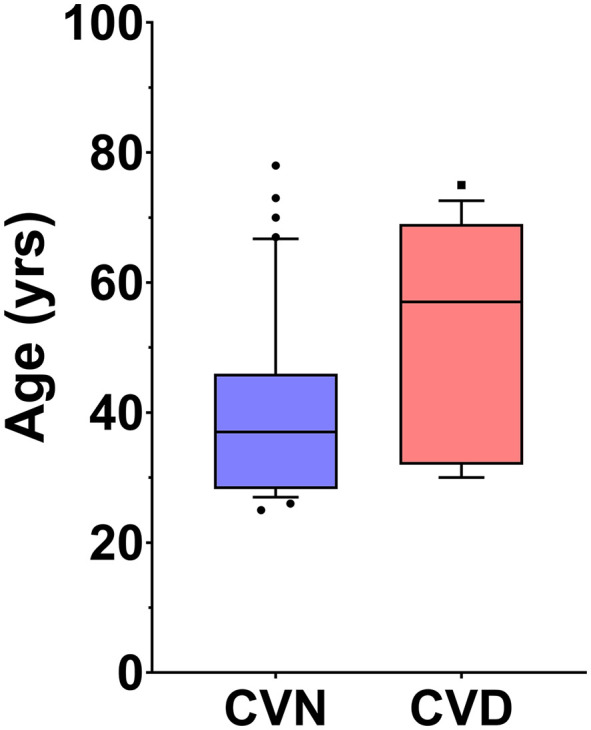
Box plots of subject age by color vision status. CVN: Color vision normal. CVD: Color vision deficient. Note that subjects with CVD are substantially older than those without CVD.

The age distribution of the 55 optometrists in our study was not normally distributed (Shapiro-Wilk = 0.889, df = 55, p < 0.001). To examine the influence of age we divided our subjects in half based on age (Tables 1, 2 and Fig 2). The age distribution within each cohort remained non-normal; therefore, non-parametric tests were used to analyze the data.

**Table 1:** Demographic characteristics of subjects by age group.

	N	Age		Sex		Color Vision	
Age Group		Mean (SD)	Range	Male	Female	Normal	Abnormal
Young (<39 y)	27	31.0 (3.8)	25-38	8	19	22	5
Old (≥39 y)	28	56.6 (12.3)	39-78	22	6	18	10

**Table 2:** Demographic characteristics of subjects by color vision.

	N	Age Group		Sex	
Color Vision		Young (<39)	Old (≥39)	Male	Female
Normal	40	22	18	16	14
Abnormal	15	5	10	14	1

Median ColorDx scores for older CVN subjects were significantly lower than those for younger CVN subjects across all three cone types (Table 3). In addition, reaction times for older CVN subjects were significantly prolonged for two of the three cone types. There was no significant difference in median ColorDx scores or response times between young and old CVD subjects.

**Table 3:** Median ColorDx results by age group and color vision status.

	Color Vision Normal			Color Vision Deficiency		
	Young	Old	p*	Young	Old	p*
Red Score	127	114	0.011	108	92.5	0.679
Green Score	120	110	0.005	55	57	0.953
Blue Score	147	129	0.019	148	113.5	0.055
Red Time^†^	1.80	3.05	<0.001	1.60	2.25	0.371
Green Time^†^	1.80	2.05	0.240	2.50	2.70	0.440
Blue Time^†^	1.05	1.70	<0.001	1.00	1.65	0.055

*Mann-Whitney U test. ^†^Median reaction time (s)

**Fig 3:**
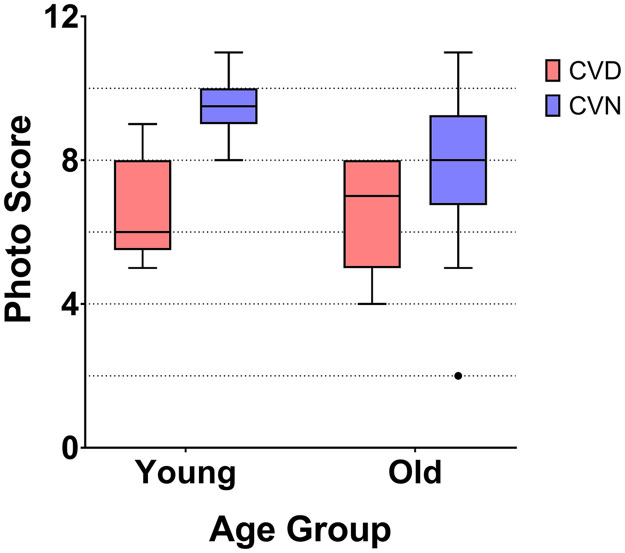
Tukey box plots of photo score by age group and color vision status. Abnormal color vision significantly decreased performance of younger but not older clinicians. CVD: Color vision deficiency, CVN: Color vision normal. Photo score: Number of correctly identified photographs.

**Fig 4:**
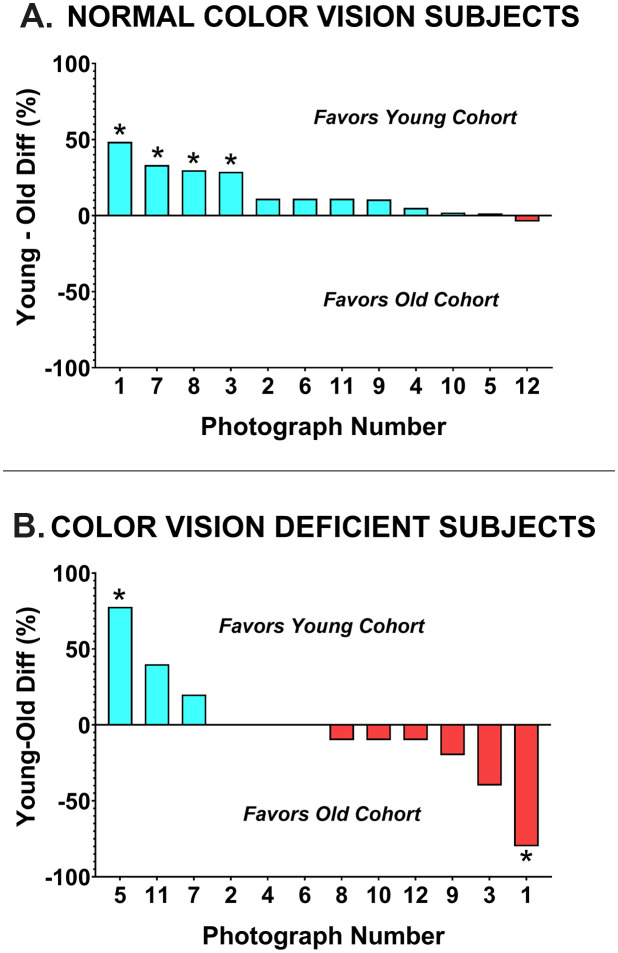
Difference in proportion of lesions correctly identified by young and old cohort of optometrists (A) Performance of subjects with normal color vision. (B) Performance of subjects with color vision deficiency. *Chi-square ≤0.05.

There was a significant decline in photo score with increasing practitioner age (r^2^ = 0.30, F(1, 52) = 21.50, p < 0.001) (Fig 1A). One less photo was correctly identified for every 15-year increase in practitioner age. Overall performances of younger and older optometrists are presented in Fig 3. The influence of age on the correct interpretation of each image was analyzed by color vision status (Fig 4). Among CVN optometrists, there was a marked difference in the number of correctly identified photographs between age groups, with younger subjects outperforming older subjects in 11 of 12 photos (Mann-Whitney U = 81.00, p = 0.001). In each of the 4 cases where there was a statistically significant difference between age groups, the younger optometrists had superior performance. The situation was very different for optometrists with CVD. The number of correctly identified photographs was identical or within 10 percentage points for 6 of the 12 photos (Mann-Whitney U = 21.00, p = 0.84). There was a statistically significant difference between younger and older CVD doctors in only 2 cases, in one case the younger doctors had superior performance and in one case the older doctors were superior.

### Influence of CVD

Red and green cone ColorDx scores for CVN and CVD subjects were normally distributed (Shapiro-Wilk p > 0.05), but blue cone scores were not. Only one subject failed on blue cone testing and this subject also failed on red and green; therefore, parametric tests were used to analyze ColorDx scores.

**Fig 5:**
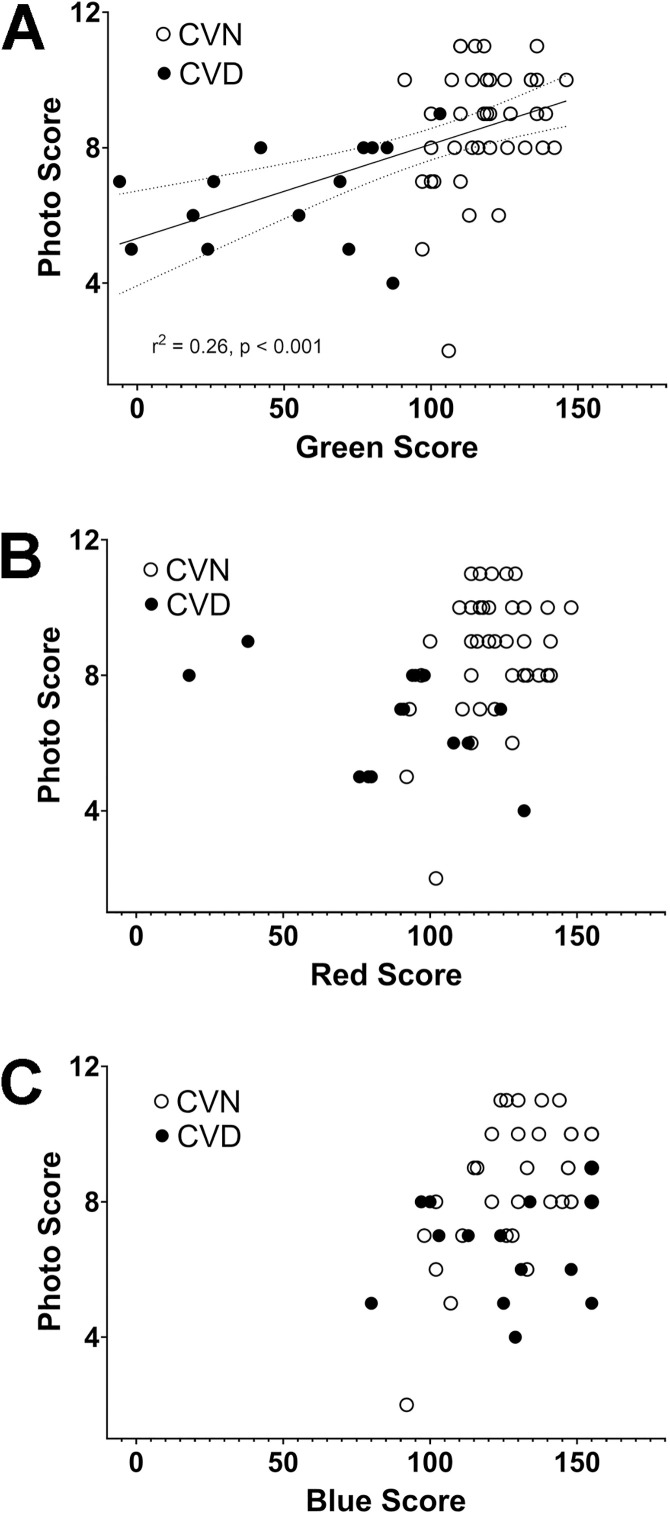
Scatter plots of photo score by ColorDx green cone score (A), red cone score (B), and blue cone score (C). CVN: Color vision normal, CVD Color vision deficiency. Photo score: Number of correctly identified photographs.

Subjects with CVD as determined by ColorDx (score <90 for any cone type) had a significantly lower mean photo score than CVN subjects (t-test, p < 0.001). As noted above, stepwise regression analysis revealed that green cone score was a significant predictor of photo score. Scatter plots of photo score by each cone type reveal that only green cone score robustly differentiates between CVN and CVD subjects (Fig 5). Linear regression revealed that for every 33-point change in green cone score photo score would change by 1 point (r^2^ = 0.26, F(1,52) = 18.26, p < 0.001).

**Fig 6:**
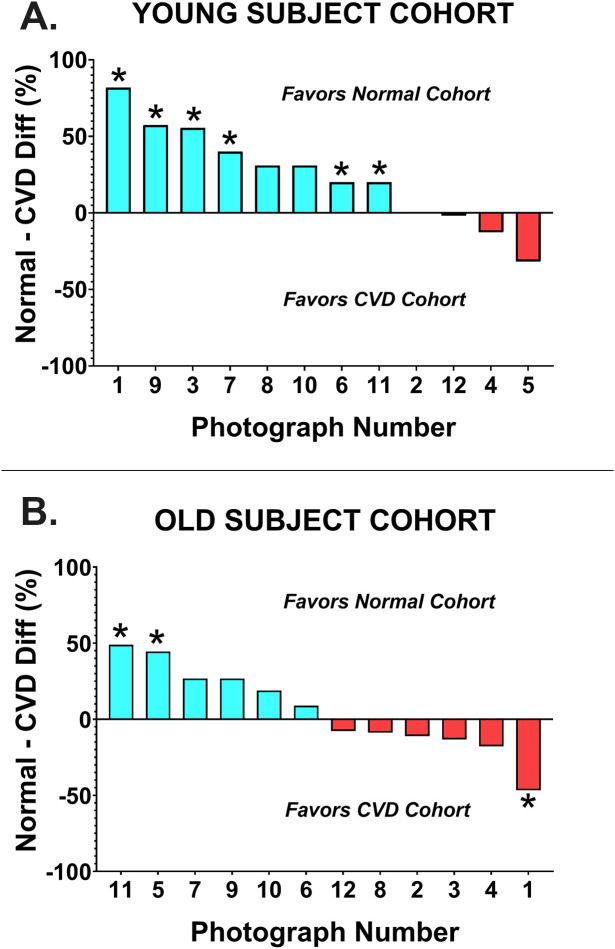
Difference in proportion of lesions correctly identified by color vision normal and color vision deficient subjects. (A) Performance of young cohort of subjects. (B) Performance of old cohort of subjects. *Chi-square ≤0.05.

The influence of CVD on the correct interpretation of each image was analyzed by age group (Fig 6). Among younger optometrists, those with CVD performed significantly worse than their CVN peers (Mann-Whitney U = 7.50, p = 0.002). Younger optometrists without CVD outperformed those with CVD in 8 of 12 photos, and the difference was significant for 6 of the photos. Of the remaining 4 cases, doctors with CVD outperformed those without in 3 cases, but the difference was not statistically significant for any of them, and 1 case was a tie.

The situation was quite different for older optometrists. There was no significant difference between older clinicians with and without CVD (Mann-Whitney U = 58.00, p = 0.23). For half the photos those without CVD had superior performance and in the other half those with CVD had superior performance. There was a statistically significant difference in 3 cases: in two cases favoring those without CVD and in 1 case favoring those with CVD.

### Influence of remediation device

Complete ColorDx data were available for 10 of the 15 subjects with CVD. While mean ColorDx scores tended to improve with use of the EnChroma remediation device, the change did not achieve statistical significance for any of the three cone types (Table 4). All subjects who initially failed the ColorDx (i.e., had at least one cone type score below 90) without EnChroma continued to fail with EnChroma. However, some improvements were observed in specific cone assessments. Specifically, when considering individual cone types, two of the individuals who failed red cone testing without EnChroma demonstrated scores above the passing threshold for that specific cone type when using the remediation device.

**Table 4:** ColorDx performance by EnChroma use^‡.^

	N	W/O ENCHROMA*	WITH ENCHROMA*	P^†^
RED SCORE	10	82.30 ± 10.23	84.80 ± 9.96	0.657
GREEN SCORE	10	39.90 ± 11.36	57.70 ± 9.11	0.156
BLUE SCORE	10	131.50 ± 6.67	127.90 ± 5.07	0.458
RED TIME^§^	10	4.24 ± 1.13	1.88 ± 0.30	0.045
GREEN TIME^§^	10	2.60 ± 0.27	1.77 ± 0.24	0.010
BLUE TIME^§^	10	1.39 ± 0.21	1.42 ± 0.16	0.855

‡Test sequence was without EnChroma first for all subjects. *Mean±SEM. † Student’s 2-sided t-test. § Reaction time (s).

Reaction times were significantly faster while wearing the remediation device for red and green cone types, but not for blue stimuli. Because the photo task was performed with the remediation device only after it was first performed without, task familiarity may account for some improvement in speed.

The photo task was completed with and without the aid of the remediation device by all 15 CVD subjects. There was no significant difference in the ability of subjects to correctly identify lesions with and without the use of the remediation device for any of the 12 photographs used in this study (Wilcoxon Signed Ranks Z = −0.144, p = 0.89).

After completing the photo task while wearing the EnChroma lenses, subjects were asked to either agree or disagree with the statement: “The remediation device I wore made it easier to identify abnormalities in the photographs.” Seven subjects disagreed or strongly disagreed with that statement, while 3 subjects agreed or strongly agreed. There was no significant difference in ColorDx scores, CBQoL scores, defect type (deutan/protan) or age group (young/old) between those who found EnChroma lenses to be helpful and those who did not.

### Impact of CVD on quality of life

Cronbach’s alpha was used to determine whether the 26 items in our modified CBQoL survey had significant shared variance. We found that among all subjects who completed the survey, Cronbach’s alpha was 0.98, indicating a very high shared variance. We therefore chose to combine all 26 survey items into a single QoL index variable by calculating the median response. We also present individual responses for the 3 optometry-specific questions that were included in the survey.

CBQoL data was analyzed for the 55 optometrists with ColorDx data. The proportion of optometrists with CVD whose QoL was adversely affected by CVD was 47% (7 of 15) for the CBQoL index, 67% (10 of 15) for identifying fundus lesions, 53% (8 of 15) for identifying eyelid lesions, and 40% (6 of 15) for judging external lesions as malignant (Table 5).

**Table 5:** CBQoL survey results^‡.^

	CBQoL Index^†^		Fundus Lesions^§^		Eyelid Lesions^¶^		Malignancy^#^	
	Normal	CVD	Normal	CVD	Normal	CVD	Normal	CVD
Moderately severe problem	0	0	0	1 (7)	0	0	0	1 (7)
Some problem	0	2 (13)	0	0	0	1 (7)	0	0
Mild problem	0	0	1 (3)	5 (33)	1 (3)	3 (20)	0	2 (13)
Hardly any problem	0	5 (33)	3 (8)	4 (27)	2 (5)	4 (27)	3 (8)	3 (20)
No problem	40 (100)	8 (53)	34 (89)	5 (33)	36 (92)	7 (47)	36 (92)	9 (60)
p*	<0.001		<0.001		0.002		0.014	
**Total**	**40**	**15**	**38**	**15**	**39**	**15**	**39**	**15**

‡Number of subjects (%). *Chi-square. †Median response to all 26 questions on the survey. Estimated degree of difficulty: §Identifying fundus lesions; ¶Identifying eyelid lesions due to color and #Judging external lesions as benign or malignant. Note: Some CVN subjects did not respond to the optometry-specific questions.

Among subjects with CVD, ColorDx scores for red and green cones tended to be lower for doctors reporting decreased QoL, but this did not achieve statistical significance. Interestingly, blue cone scores moved in the opposite direction. Blue cone scores were significantly higher for CVD subjects reporting decreased QoL compared to CVD subjects not reporting any difficulties associated with their CVD (t-test, F = 0.132, df = 13, p = 0.002).

There was no significant difference in either the merged CBQoL index variable or the 3 optometry-specific survey questions between the young and old optometrist cohorts.

## Discussion

This study investigated the influence of CVD on the ability of optometrists to correctly identify lesions in digital color fundus photographs. While many studies have documented the adverse effect of CVD on general medical practice [9,18,19], to our knowledge only one has investigated the impact of CVD in eye care. AlRyalat, et al. [12] modified digital color photographs of diabetic retinopathy to simulate the effect of CVD. They presented 150 such photographs to a retinal specialist who graded the retinopathy and evaluated the presence of macular edema. They found that simulated CVD decreased the accuracy of retinopathy and macular edema assessment, especially for protanopic defects. Our research is the first, to our knowledge, to study eye care professionals with CVD performing a clinical task.

Our results indicate that both age and CVD influence the ability of optometrists to correctly identify lesions in fundus photographs. Among optometrists with normal color perception, younger clinicians (<39 years of age) correctly identified lesions more frequently than older clinicians (≥39 years of age) in 11 of 12 photographs, and this was statistically significant for one-third of the images. Stepwise regression analysis revealed that reaction time to blue stimuli was a stronger predictor of performance on the photo task than age. It is well known that reaction times slow with age [20]. This is due, at least in part, to older adults prioritizing accuracy of responses over speed. Hence, it is not surprising that we found strong correlations between older age and slower reaction times. Among younger subjects slower reaction times may indicate that the individual is having more difficulty making a decision or may be less confident in their responses. Hence, reaction time may be a superior predictor of photo score because it captures age-related and age-independent factors influencing photo score. While all three reactions times where significantly correlated with age, the relationship was strongest for blue stimuli. Furthermore, only blue reaction time was significantly correlated with photo score.

There are several possible reasons why age may influence the ability to correctly identify fundus lesions in photographs. One is that age may be a surrogate for duration of time since graduation from optometry school. The superior performance of more recent graduates is perhaps attributable to greater emphasis that optometry schools place on training in ocular disease today than in the past [21,22]. Another is that age-related changes in vision may adversely affect the ability to clearly see and identify lesions. While all subjects in this study had 20/30 or better visual acuity at near, many age-related ocular changes may adversely affect the quality of vision, including dry eye, increase in optical aberrations, reduced contrast sensitivity and neurodegeneration [23–26]. We found that color perception, as quantified by ColorDx cone scores, was significantly reduced among our older CVN subjects relative to the younger CVN cohort. This is consistent with the findings of Iizuka and colleagues that found an age-related decline in ColorDx scores among CVN subjects [27]. They attributed this decline to normal age-related processes such as yellowing of the crystalline lens and other factors which may impair color perception. This may, in part, account for the poorer performance of the older CVN subjects in this study.

The presence of CVD had a significant detrimental effect on the ability of optometrists to correctly identify fundus lesions in color photographs. This finding is consistent with prior reports that CVD has an adverse effect on clinical tasks [8]. This indicates a need for interventions that have the potential to improve the ability of CVD clinicians to recognize fundus lesions. Currently, there are no effective means of improving the color perception of CVD individuals. However, efforts are being made to assist medical trainees overcome their color vision deficiency. For example, Rubin, et al. [28] report some success using color overlays, tinted contact lenses, and grayscale images to teach histology to CVD medical students. While the EnChroma lenses used in this study did not significantly improve the performance of CVD subjects, it made the task of identifying fundus lesions qualitatively easier for several individuals. Optometry students with CVD may benefit from being introduced to such aids early in their training.

Other studies have attempted to evaluate the relative influence of protan and deutan CVD on various performance measures. Elfalah et al. [29], using photographs of benign and malignant pigmented skin lesions that were manipulated to simulate CVD found that only deutan-simulated images resulted in decreased accuracy in classifying lesions as benign or malignant. However, AlRyalat et al. [12], using photographs of diabetic retinopathy that were manipulated to simulate CVD found that retinopathy classification accuracy was lowest for protan-simulated images. We found no significant performance difference between deutan and protan subjects for any of the 12 fundus photos. However, it is important to note that the present study included a limited number of subjects with specific CVD subtypes (e.g., only 3 protan subjects), which restricts the study’s ability to draw definitive conclusions about the differential impact of protan versus deutan defects on lesion identification. Further research with larger, more balanced cohorts is needed to thoroughly investigate these potential performance differences.

We found that the EnChroma Indoor lenses used in this study produced no significant change in ColorDx scores or performance on the lesion identification task by CVD subjects. Red and green reaction times were, however, significantly faster with the remediation device. Konan suggests response times be considered a secondary measure of performance and may represent a measure of the subject’s confidence in their response. Hence, it is possible that the remediation device offered CVD subjects some visual cues that resulted in greater confidence in their responses. However, because our subjects always completed the photo task while wearing EnChroma lenses after they first performed the task without the lenses, the more rapid response times might also reflect familiarity with the task rather than greater confidence in their responses. A majority of subjects reported that the lenses produced no subjective improvement in their ability to identify lesions in fundus photographs. These findings are consistent with prior reports that commercially available remediation devices do not provide clinically significant improvement in color perception [15]. However, it is interesting to note that several optometrists reported that the lenses did make identifying lesions easier, even though their performance did not significantly improve. Hence, trial use of such lenses by CVD clinicians may be worthwhile to determine whether they subjectively experience any improvement in clinical practice. It should also be noted that duration of use may influence the effect of remediation devices [30].

A QoL index composed of the median response to the 26-item modified CBQoL survey showed that almost half (47%) of optometrists with CVD reported difficulties in their daily activities attributed to poor color perception. There was no significant difference in CBQoL responses between younger and older CVD optometrists. This is consistent with our finding that there was little difference between younger and older CVD optometrists in correctly identifying lesions in fundus photographs. This suggests that the adverse impact of CVD is stable over time. The severity of CVD was not significantly worse among optometrists with and without a reported decrease in QoL, suggesting that the effect of CVD on QoL may not directly relate to the severity of the CVD. This is in contrast with other reports suggesting that individuals with milder color vision loss experience fewer problems [8,31,32]. This discrepancy may be due to our small sample size and differences in how severity of CVD was assessed. Cole acknowledges that color vision tests are not “perfect predictors” of who will have problems and who will not [3]. We found that mean blue cone scores were significantly higher among CVD subjects reporting decreased QOL compared to CVD subjects that did not report a decrease in QOL. While this could be an artifact of our small sample size, another intriguing possibility is that blue light sensitivity is indeed related to QOL impairment, possibly through its known association with melatonin suppression [33,34]. We are unaware of any prior studies that have examined the relationship between cone contrast thresholds and QOL. Regarding the 3 questions that specifically pertain to ophthalmic practice, optometrists with CVD reported a significantly higher level of difficulty for each task compared to optometrists without CVD. Taken together, these findings suggest that CVD may pose a disability for eye care providers that is not directly related to the severity of loss and does not improve with practice experience.

Our findings agree with prior research indicating that CVD has a significant adverse effect on CBQoL measures. Gao and Tian [6] investigated the impact of CVD among Chinese college students using the CBQoL instrument. They found that 68% of 312 students reported a negative effect of CVD on their QoL. Male, et al. [7] using a modified version of the CBQoL questionnaire investigated QoL among 60 patients with congenital or acquired CVD at two eye hospitals in India. They found that 100% of individuals with CVD had abnormal QoL scores. Both studies report higher proportions of subjects with CVD suffering impaired QoL than we found (47% of affected optometrists). This may be due to the very different populations involved in these studies. Perhaps optometrists cope differently with the adverse effects of CVD by virtue of their vision science background. Conversely, students and other lay people may be more challenged by abnormal color vision, especially when it is acquired.

Strengths of this study include assessment of clinicians with CVD rather than employing simulated images and the quantitative assessment of color vision using the ColorDx system. Limitations of this study include the small number of subjects with CVD. Another factor that may influence interpretation of our results were the significant interactions of age, sex, and CVD among our subjects. Those subjects with CVD were significantly older than those without CVD. In addition, the older cohort of subjects were predominantly male while the younger cohort were predominantly female. Therefore, the performance penalty of CVD found in this study may not accurately reflect the disability imposed by CVD on the practice of optometry. Additional research is needed to better understand the degree to which CVD impacts the clinical performance of optometrists and other eye care providers.

## Conclusions

The study findings indicate that both congenital color vision deficiency (CVD) and older age are factors that can decrease the ability of optometrists to correctly identify lesions in fundus photographs and hence may increase the risk of errors in diagnosis and treatment. This has led many commentators to recommend that individuals entering medical practice undergo color vision testing and counseling if a deficiency is found [18,35–38]. Given that 27% of the optometrists with CVD in this study were previously unaware of their condition, we endorse this recommendation.

## Supporting information

S1 File Fundus photographs. (ZIP)
