# Supplementary figures and images for: Effect of congenital color vision deficiency on the ability of optometrists to correctly identify lesions in ocular fundus photographs
Source: PLoS One. 2025 Nov 24;20(11):e0337626. doi: 10.1371/journal.pone.0337626 (PMC12643291; doi:10.1371/journal.pone.0337626)

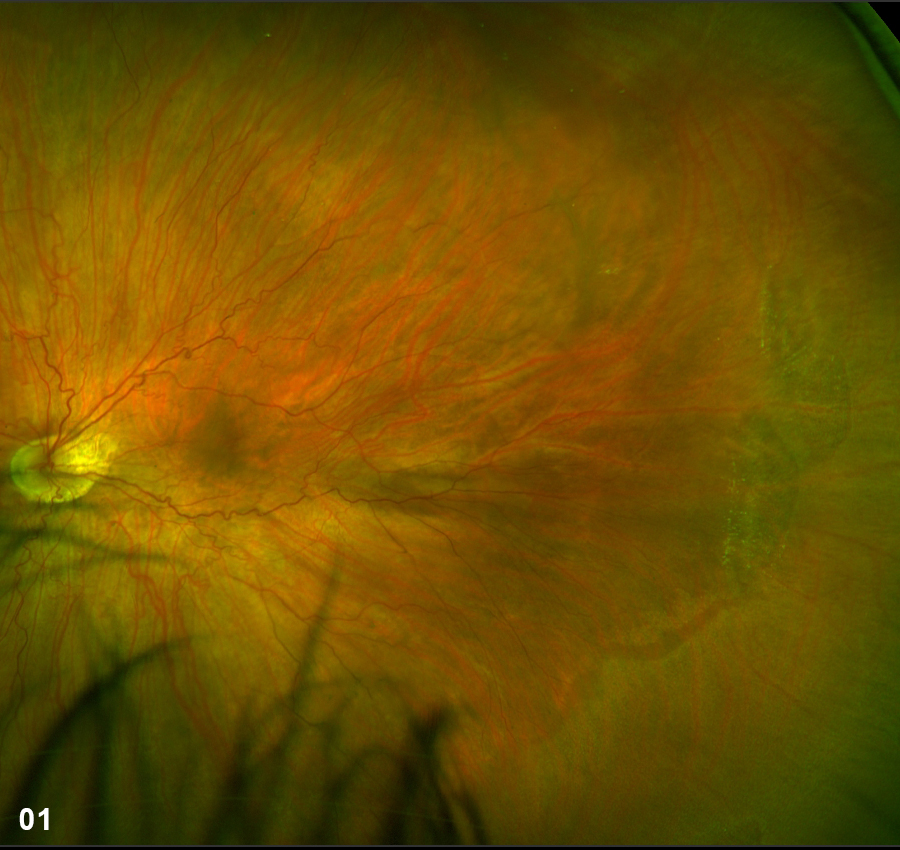

Supplement: S1 File — (ZIP) [file pone.0337626.s001.zip › 01.jpg]

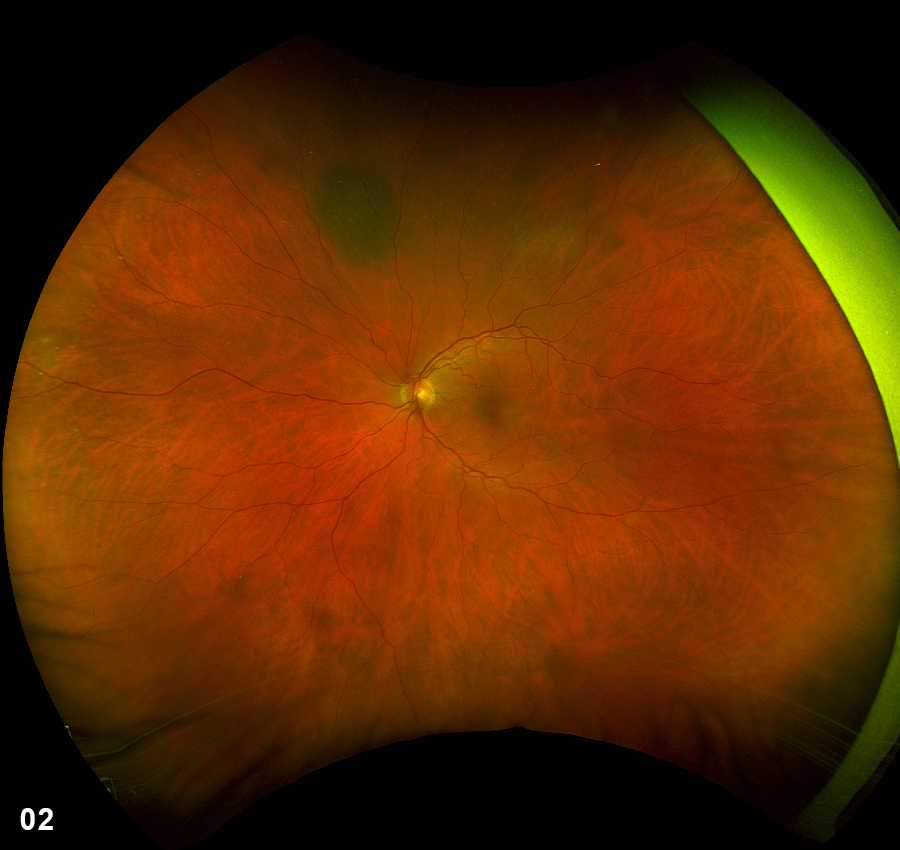

Supplement: S1 File — (ZIP) [file pone.0337626.s001.zip › 02.jpg]

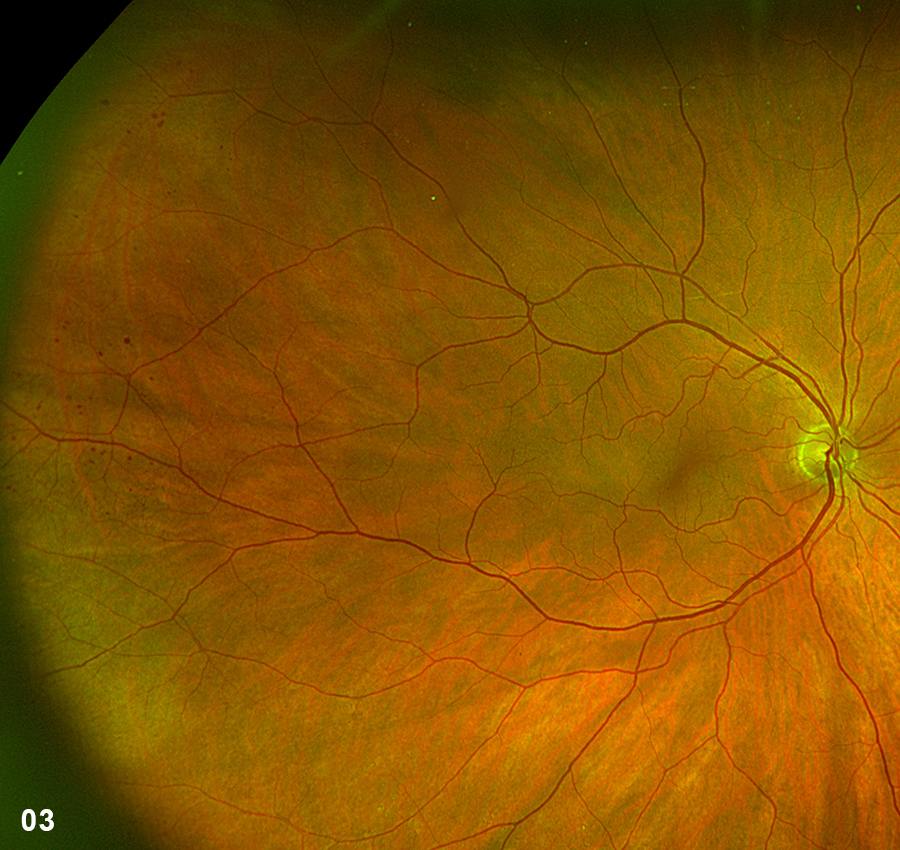

Supplement: S1 File — (ZIP) [file pone.0337626.s001.zip › 03.jpg]

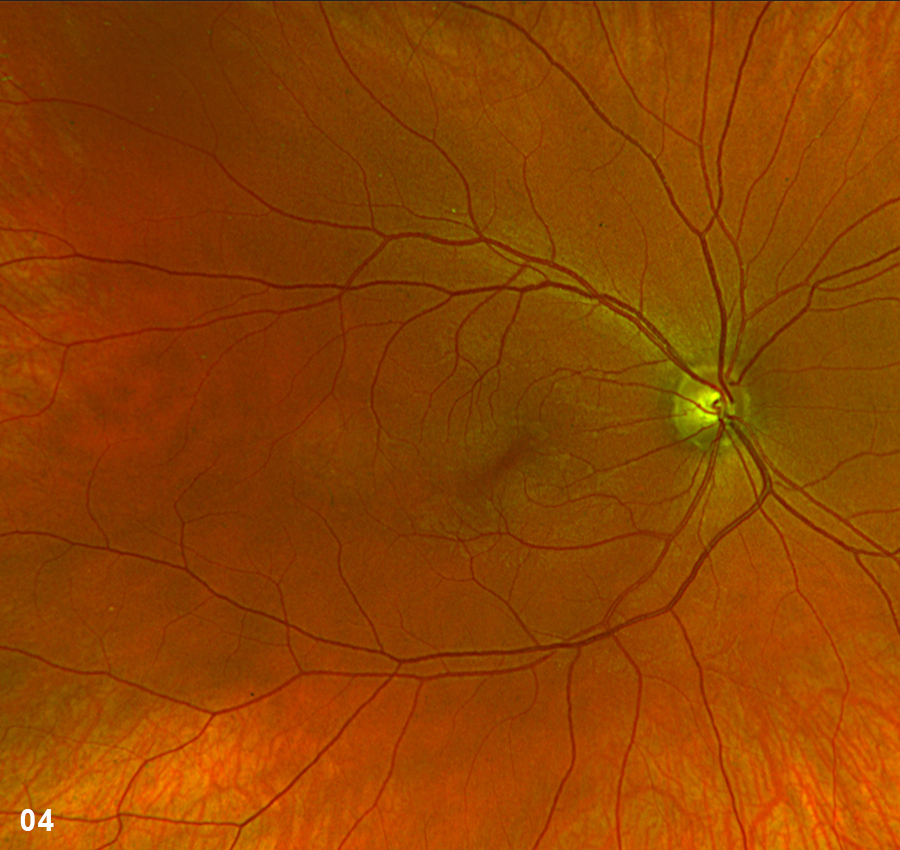

Supplement: S1 File — (ZIP) [file pone.0337626.s001.zip › 04.jpg]

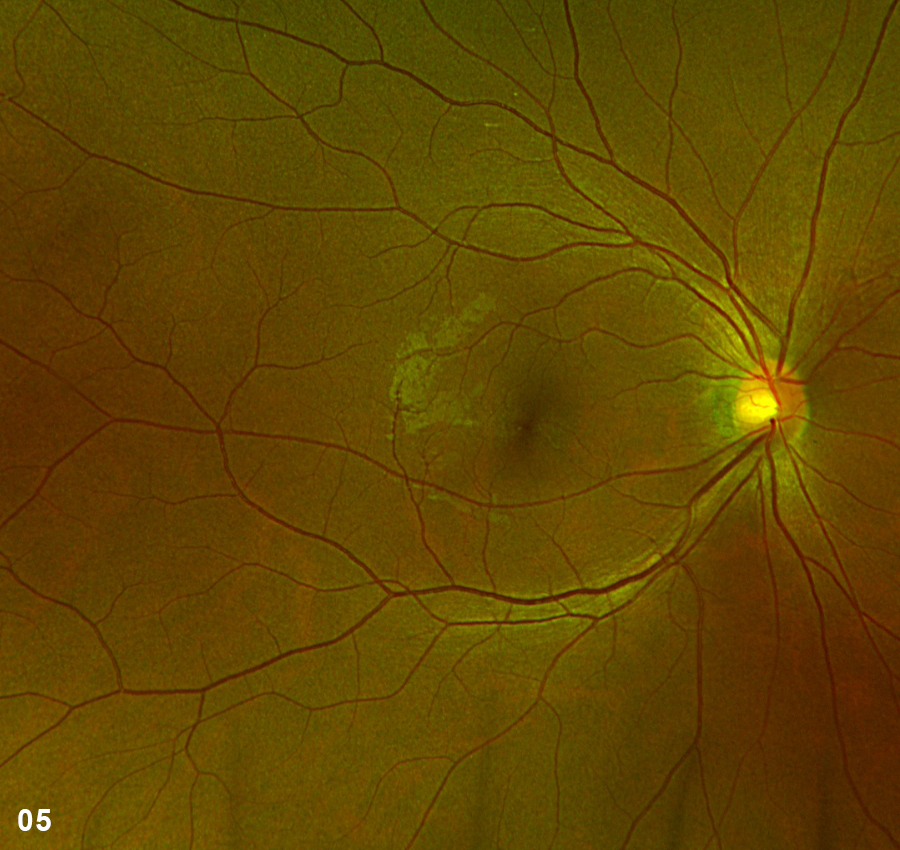

Supplement: S1 File — (ZIP) [file pone.0337626.s001.zip › 05.jpg]

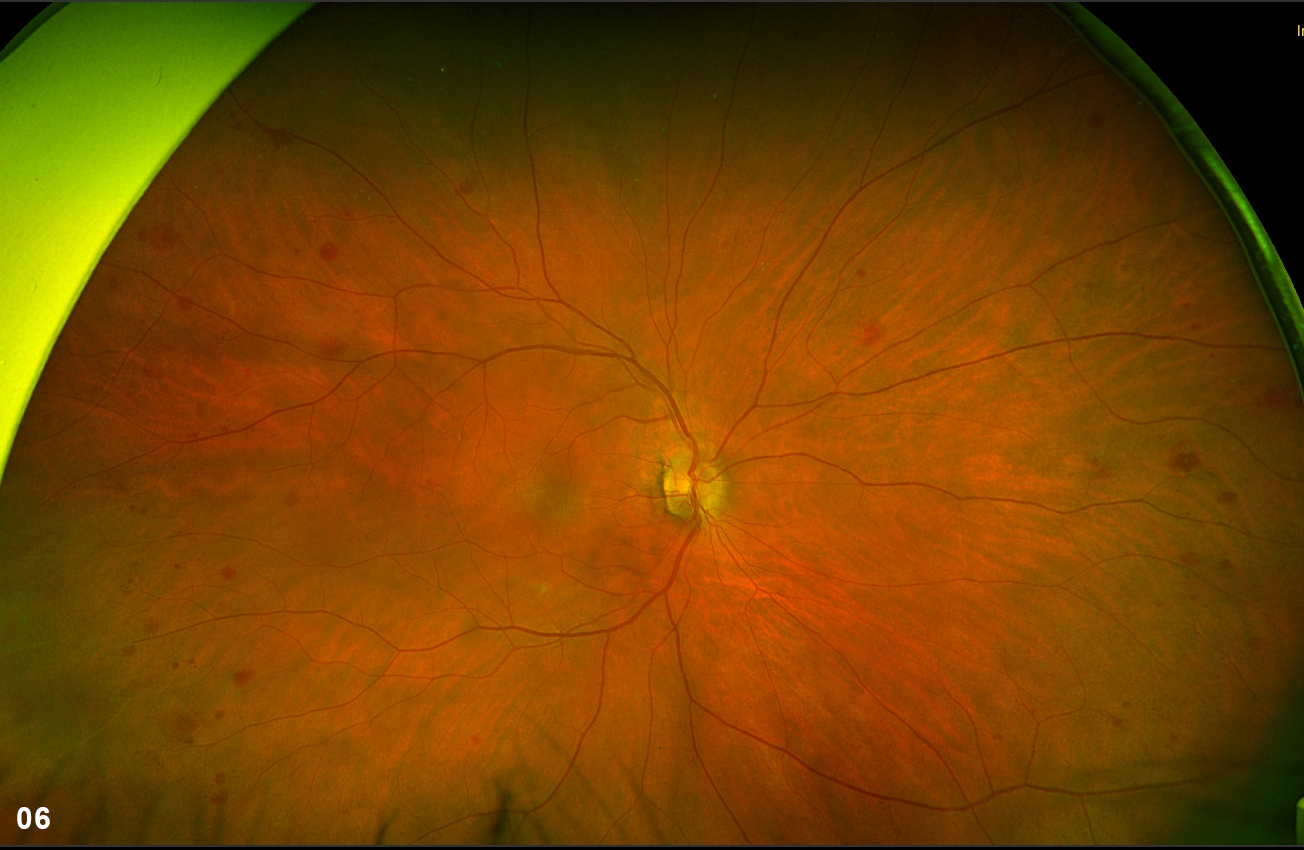

Supplement: S1 File — (ZIP) [file pone.0337626.s001.zip › 06.jpg]

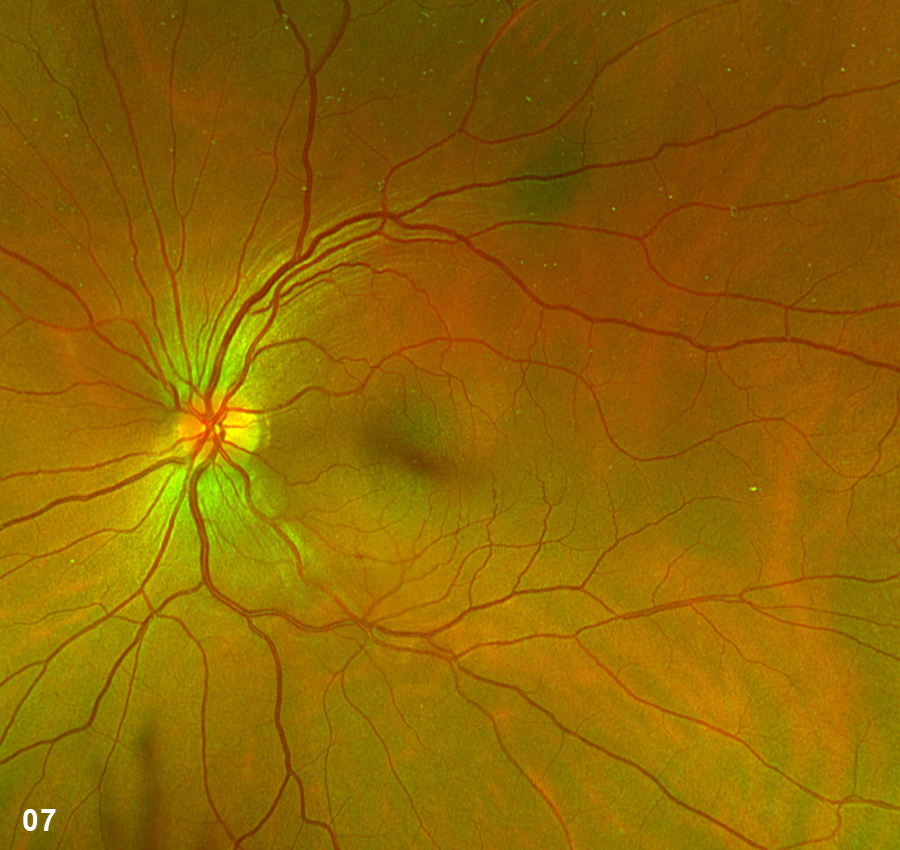

Supplement: S1 File — (ZIP) [file pone.0337626.s001.zip › 07.jpg]

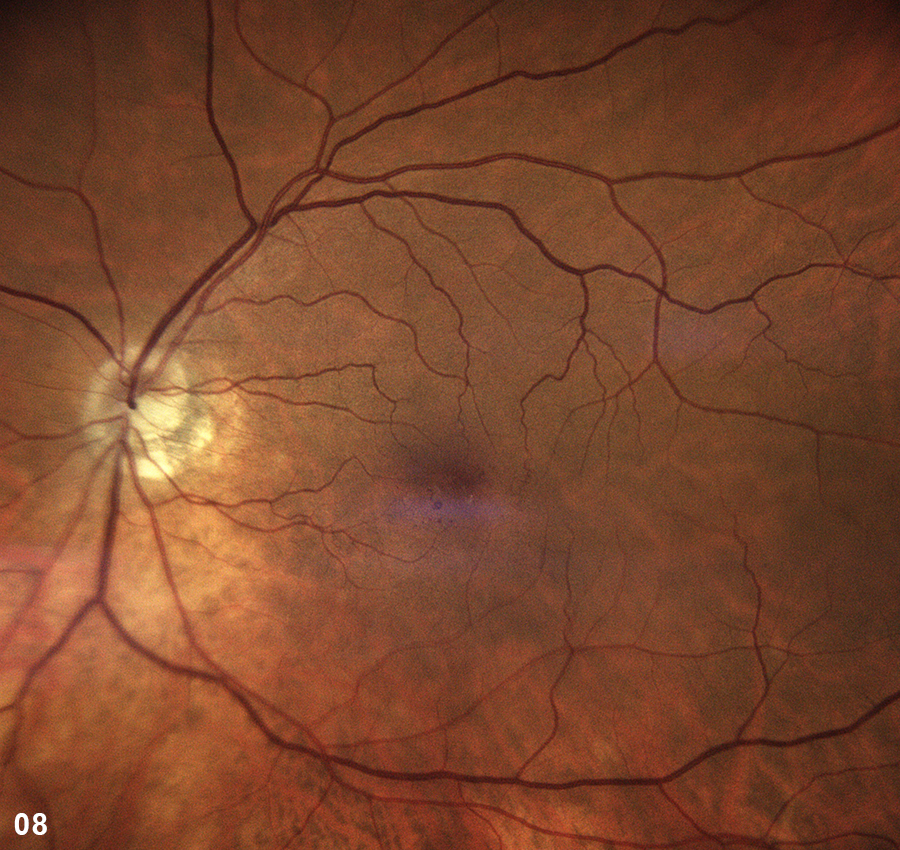

Supplement: S1 File — (ZIP) [file pone.0337626.s001.zip › 08.jpg]

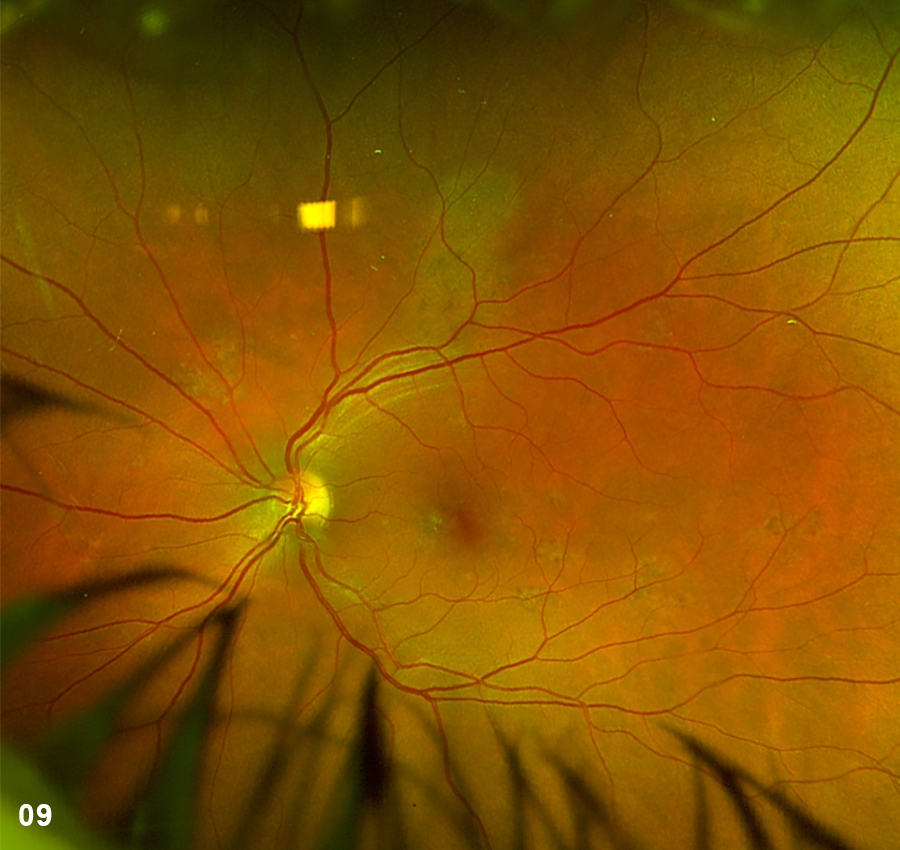

Supplement: S1 File — (ZIP) [file pone.0337626.s001.zip › 09.jpg]

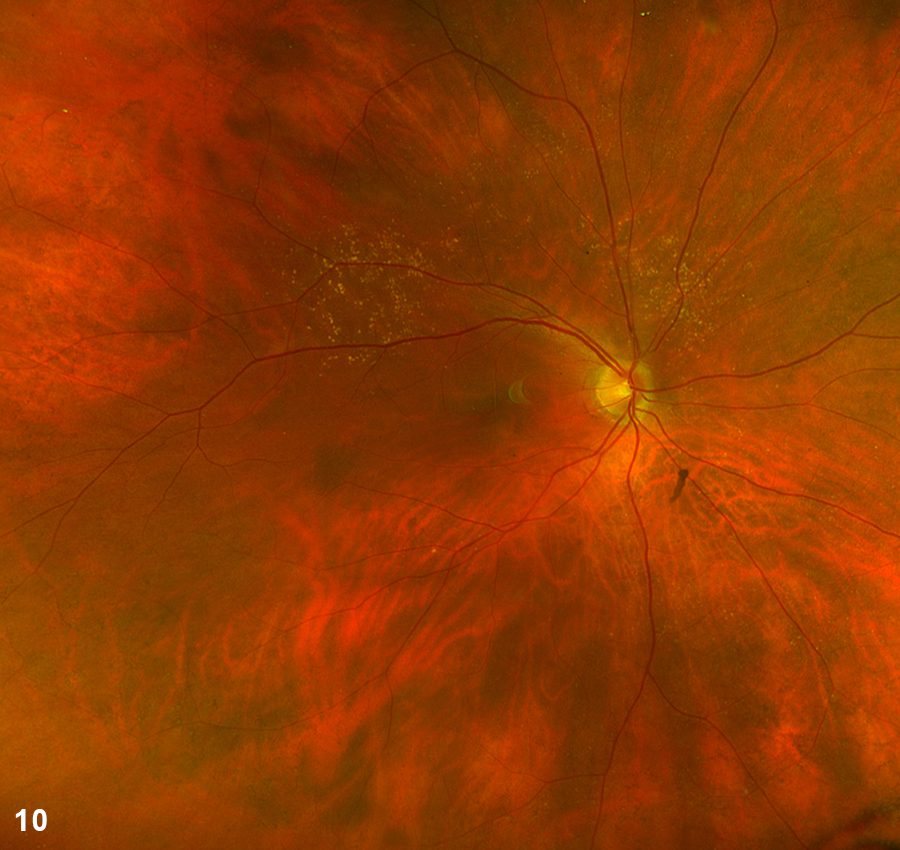

Supplement: S1 File — (ZIP) [file pone.0337626.s001.zip › 10.jpg]

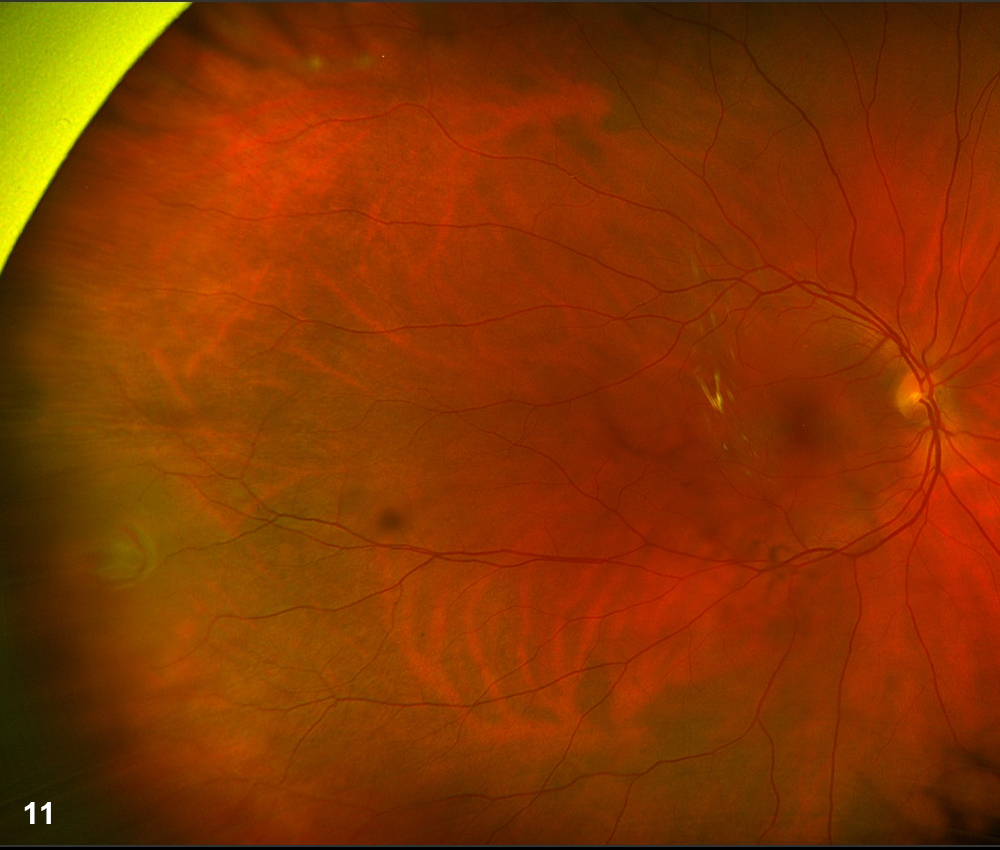

Supplement: S1 File — (ZIP) [file pone.0337626.s001.zip › 11.jpg]

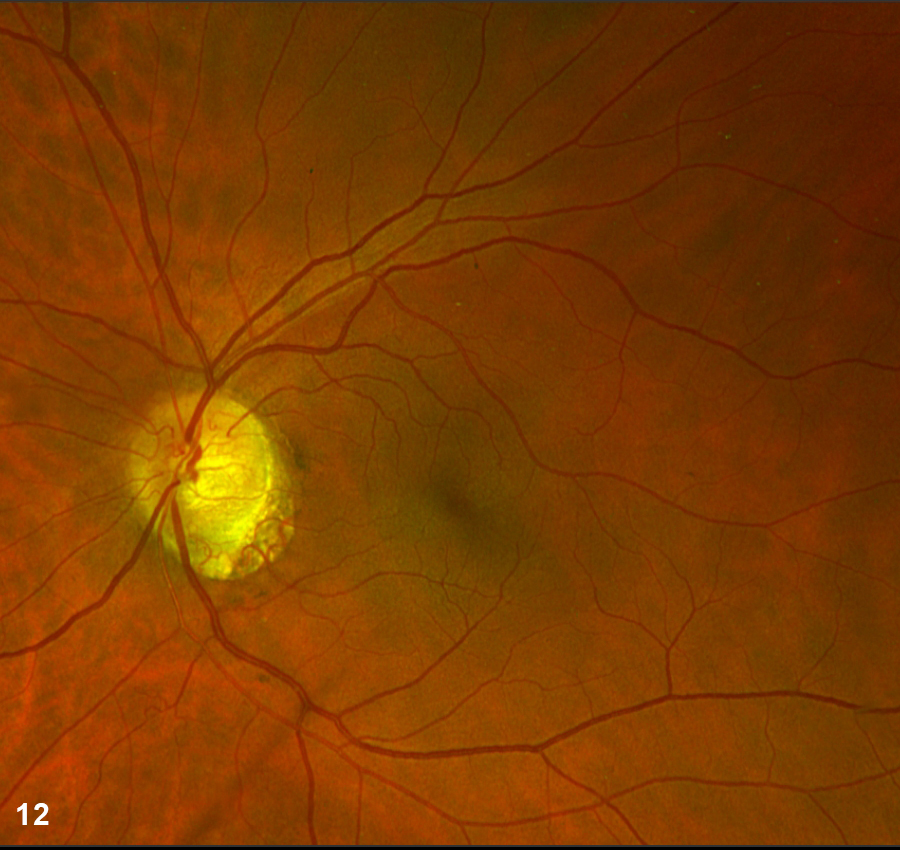

Supplement: S1 File — (ZIP) [file pone.0337626.s001.zip › 12.jpg]
